# Norm scores of cancelation and bisection tests for unilateral spatial neglect: data from a Brazilian population

**DOI:** 10.6061/clinics/2019/e1468

**Published:** 2020-04-30

**Authors:** Gustavo José Luvizutto, Marcelo Ortolani Fogaroli, Rodolfo Mazeto Theotonio, Eduardo de Moura Neto, Hélio Rubens de Carvalho Nunes, Rodrigo Bazan

**Affiliations:** I Departamento de Fisioterapia Aplicada, Universidade Federal do Triangulo Mineiro (UFTM), Uberaba, MG, BR; II Neurocirurgia, Faculdade de Medicina de Botucatu, Universidade Estadual Paulista (UNESP), Botucatu, SP, BR; III Servico de Atencao e Referencia em Alcool e Drogas (SARAD), Hospital das Clinicas, Faculdade de Medicina de Botucatu, Universidade Estadual Paulista (UNESP), Botucatu, SP, BR; IV Pos Graduacao em Educacao Fisica, Biomecanica e Controle Motor, Universidade Federal do Triangulo Mineiro (UFTM), Uberaba, MG, BR; V Departamento de Saude Publica, Universidade Estadual Paulista (UNESP), Botucatu, SP, BR; VI Departamento de Neurologia, Psicologia e Psiquiatria, Faculdade de Medicina de Botucatu, Univesidade Paulita (UNESP), Botucatu, SP, BR

**Keywords:** Diagnosis, Unilateral Spatial Neglect, Standardization, Line Bisection Task, Line Cancelation Task, Star Cancelation Task

## Abstract

**OBJECTIVE:**

Unilateral spatial neglect (USN) results in a consistent and exaggerated spatial asymmetry in the processing of information about the body or space due to an acquired brain injury. There are several USN tests for clinical diagnosis, but none of them are validated in Brazil. The aim was to obtain normative values from a healthy sample in Brazil and to evaluate the effects of demographic variables on USN tests.

**METHODS:**

This was a cross-sectional study performed with 150 neurologically healthy individuals. USN was evaluated using the line cancelation (LC), star cancelation (SC), and line bisection (LB) tests in the A3 (29.7 x 42.0 cm) sheet format.

**RESULTS:**

In LC, 143 participants had 0 omissions, and the occurrence of failure was significantly associated with aging (OR=1.1[1.02-1.2]; *p*=0.012). In SC, 145 participants had fewer than 1 omission, and the occurrence of failure was significantly associated with aging (OR=1.07[1.03-1.11]; *p*<0.001). In LB, deviations were the lowest for those with the highest level of education (r=0.20; *p*=0.015), and the deviation was 9.5 mm.

**CONCLUSION:**

The cutoff points presented in this study may be indicative of USN, but due to performance differences based on age, we suggest using different norm scores for different age groups. These norm scores can be used in the clinic immediately for USN diagnosis.

## INTRODUCTION

Unilateral spatial neglect (USN) results in a consistent and exaggerated spatial asymmetry in the processing of information about the body or space due to an acquired brain injury that cannot be accounted for by either sensory or motor deficits 1-4. Often, USN is associated with lesions in the right hemisphere, involving mainly cortical regions—such as the superior and middle temporal gyri, inferior parietal lobule, insula, inferior frontal gyrus and the Rolandic operculum—and subcortical regions—such as the basal ganglia—as well as the white matter fiber tracts of the superior longitudinal fasciculus, superior occipitofrontal fasciculus and inferior occipitofrontal fasciculus 3,5-7.

USN is associated with lower functional performance and is a major contributor to the slowing of neurological recovery 8-10. To diagnose USN, standard tests are required, and several tests should be used in the evaluation. USN is commonly assessed in the clinic using either the line bisection (LB) task or the target cancelation task 11. USN tests were first proposed by Albert in 1973 12. During these tests, the patient is asked to find and cancel random lines on a sheet of paper. This is the line cancelation (LC) task, and it is sensitive to spatial asymmetry in the processing of information about the body or space 13.

Performance on visuospatial tasks may change with current stimulations and, possibly, with task demands. Several studies have reported that the presence of distractors, such as in target-versus-distractor distinctions, can induce more neglect in cancelation tests 14. Wilson et al. proposed the star cancelation (SC) test, which uses nontarget distractor stimuli during the test 15. The authors of this study concluded that the SC test may be more sensitive than the LC test for USN evaluation. Another test used to evaluate USN is the LB test, during which participants are asked to find the midpoint of a horizontal line displayed on a sheet of paper. The LB test is sensitive regarding USN detection in individuals with right hemisphere lesions 11.

Commonly, the USN tests are administered as paper-and-pencil tests in the A4 sheet format 16. Although several tests are described in the literature, none have provided normative data using a large paper format. We hypothesize that using the larger A3-format paper better reflects the interaction between the individual and the environment. The organization of actions is specific to the task and the environment in which the task is being performed 17, and the A3 format, provides a larger task exploration field.

The only test validated in Brazil in recent years is the face-hand test, which measures sensorial extinction or sensorial neglect, but no norm scores have been collected for pen-and-paper tests 13. Neuropsychological tests, including visual tests, can be influenced by demographic characteristics, such as age and education 18. Several studies have shown that factors such as individual reading habits, cognitively demanding activities and, especially, higher educational levels can influence cognitive capacity and increase performance in visuospatial tasks 19-22. Standardizing these clinical tests and collecting norm scores in a nonclinical sample will provide parameters for normality comparisons and can help professionals involved in the rehabilitation process objectively diagnose USN and propose appropriate interventions. The aims of this study were 1) to examine the effects of age, sex and level of education on performance on the three USN tests and 2) to provide norm scores that can be used in the clinic, split for age, sex and/or level of education in case these variables affect performance.

## MATERIALS AND METHODS

The participants in this cross-sectional study were neurologically healthy individuals, such as graduate students of the Botucatu Medical School (UNESP), professionals at the Clinical Hospital of Botucatu Medical School and patients hospitalized in the orthopedics, urology and vascular units. The participants were recruited to participate in the study from January to December 2016 via direct contact from the researcher.

The study was approved by the Ethics in Human Research Committee (number 122/2011). Our inclusion criterion was being aged 18 years or older, and our exclusion criteria were not being aware of the tests they performed (Glasgow coma scale <14); being hemodynamically unstable (defined as 1 or more out-of-range vital sign measurements); having a history of neurological disorder (evaluated by medical records); having a history of substance abuse or dependence (as assessed by history, record review, and serum toxicology); using medications with central nervous system effects; having a history of learning disability; and scoring 20 points or less on the Mini Mental Status Examination (MMSE) for individuals with 1 to 4 years of education, scoring <25 points for individuals with 5 to 8 years of education, scoring <26.5 points for individuals with 9 to 11 years of education, and scoring <29 points for individuals with more than 11 years of education 23. Participants had no signs of discomfort at the time of USN evaluation.

### Variables

We obtained and analyzed information on the following demographic variables from the study participants by direct interview: age (years), sex (male or female), years of education and ethnicity. We evaluated the effects of age, sex and years of education on neglect scores, and ethnicity was used to describe the sample characteristics.

In all USN tests, the examiner used A3 paper (29.7 x 42.0 cm), centered in front of the patient so that there was a distance of 50 cm from the glabella to the center of the paper 14.

We used well-established methods for the cancelation and bisection tests 24.

### a) Cancelation Tests

- Line cancelation (LC) test: The test was carried out using an A3 sheet containing 40 lines with a length of approximately 2.5 cm. The lines were drawn in 6 different orientations. The sheet contained 18 lines on each side (right or left) and 4 lines at the midline. The examiner asked the following question of the subject once the test was finished: “Have all the lines been crossed?” The test was terminated when the answer was affirmative. If the answer is negative, the test continued. The total omission score was the proportion of lines omitted relative to the total number of lines, and left-right differences were also calculated 12,24. There was no time limit for performing the LC test.

- Star cancelation (SC) test: The test was carried out using an A3 sheet containing 52 large stars, 13 letters, and 10 words randomly interspersed with 56 smaller stars. The individual was asked to find and cross out (cancel) only the smaller stars after the examiner demonstrated the procedure by striking out two stars in the center of the sheet. The total omission score was the number of omitted stars subtracted from the total number of stars presented in the test, and left-right differences were also calculated 15,24-25. There was no time limit for performing the SC test.

### b) Line Bisection Test

- Line bisection (LB) test: Individuals were presented with 18 horizontal lines arranged in six rows of three columns (right, center, and left) on an A3 sheet, with a line at the upper end of the sheet. The lines were 200 mm long and 1 mm in width and were organized in different positions. Individuals were asked to place a mark through the center of a series of 18 horizontal lines with a pencil. After the test was completed, we determined the value, in millimeters, of the scratched portion in relation to the rest of the line using the formula:



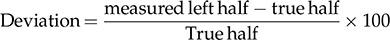



This transformation yields positive numbers for marks placed to the right of the center and negative numbers for marks placed to the left of the center 11,24,26. This formula was used for each line, and we also analyzed the “mean value of deviation” (MVD), which was obtained by adding all of the deviations and dividing the resulting value by the total number of test lines.

### Sample Size Calculation

The hypothesis was based on the association between age, sex, level of education and chance of failure on USN tests. Therefore, assuming simple random sampling and type I and II errors equal to 0.05 and 0.25, respectively, the chance of failing the USN tests are 0.17 and 0.05, for participants with low and high education, respectively. Accordingly, the minimum sample size was estimated to be 135 participants, with this number increased by 10% to offset information loss.

### Statistical Analyses

The Shapiro-Wilk test rejected the hypothesis of normality for all numerical variables in the study, and the kurtosis (k) was also calculated for variability analysis. Costa Neto reports that symmetrical distributions have k values of 3, while leptokurtic (asymmetric) distributions have k values greater than 3 27. Therefore, we estimated odds ratios (the chance of an event occurring in a group) for the number of omissions in the LC and SC tests by a linear regression model based on demographic variables (age, sex and education). The association between LB values and sociodemographic variables was investigated using the Mann-Whitney test and Spearman's correlation. All associations and areas under the ROC curve were treated as significant if *p*<0.05. Analyses were performed using SPSS software (version 21.0, SPSS, Chicago, IL, USA).

## RESULTS

We evaluated and screened 250 individuals, but only 150 met the inclusion criteria for the study (hemodynamically unstable=19; loss of consciousness=24; MMSE <20 points for individuals with 1 to 4 years of education=26; MMSE <25 points for individuals with 5 to 8 years of education=19; MMSE <26.5 points for individuals with 9 to 11 years of education=10; and MMSE <29 points for individuals with more than 11 years of education=2). The demographic characteristics and performance of individuals in the USN tests are presented in Table 1.

The estimated odds ratios for the number of omissions in the LC and SC tests based on demographic variables showed no association with any sociodemographic factors except age (Table 2). Table 2 shows that the chance of failure in the LC test was significantly associated with age (OR=1.1 (1.02 to 1.2); *p*=0.012), and the chance of failure in the SC test was also significantly associated with age (OR=1.07 (1.03 to 1.11); *p*=0.000).

Table 3 shows that the deviation from the center was the smallest among individuals with the highest education levels (r=-0.20; *p*=0.015). We also observed a larger variance in women than in men. Specifically, the kurtosis was higher among women (k=13.6) than among men (k=3.3), demonstrating greater variability and asymmetry of data among women. In the LB test, we observed median deviations from the center of 6.2 with a range of 5.8 to 6.6 mm using a 95% confidence interval.

We observed a significant correlation between age and level of education (r=-0.40, *p*<0.001; Spearman). For this reason, we decided to stratify the USN tests only by age group. Table 4 shows the results of each USN test expressed in terms of the mean, standard deviation and percentile and stratified by age.

Table 5 shows a summary of performance on the paper-and-pencil USN tests. In the LC test, we used values above 0 for USN classification. In the SC test, we used >0 omissions in individuals up to 35 years old, >2 omissions in individuals between 36 and 60 years old, >3 omissions in individuals over 60 years old and >1 omission in general for USN diagnosis. In the LB test, we used >8 mm in individuals up to 60 years old, >13 mm in individuals 61 to 65 years old and >17 mm in individuals over 65 years old, and >9.5 mm in general.

## DISCUSSION

This study aims to establish normative values for three USN tests printed on A3 paper for a Brazilian population without neurological disorders. The individuals in our study demonstrated a higher frequency of omissions in the SC test than in the LC test, as described previously 24. The stimuli presented in the SC test are more dispersed, and performance on visuospatial tasks may change with task demands.

The present study found that participants made errors in the tasks that measured spatial exploration and established the cutoff point for USN tests. The presence or absence of USN in LC, defined by Albert, is based on the number of lines left uncrossed on each side of the test sheet 12. If any lines are left uncrossed and more than 70% of uncrossed lines are on the side contralateral to brain injury, USN is indicated. In our study, we required all lines in the LC test be crossed to rule out USN.

Halligan et al. reported that the maximum score that can be achieved on the SC test is 54 (56 small stars in total, minus the 2 used for demonstration), and a cutoff of 51 is used to diagnose USN 28-30. Other authors have observed that the SC test can be influenced by distractors that hinder attention, and the test is indicated for diagnosing mild cases of USN 15,24,31. In our study, we required the participants to cross 53 stars in the SC test to rule out USN.

Cancelation tasks that employ a random arrangement of complex symbols are more difficult and hence more sensitive for detecting neglect than similar tests that are arranged in organized rows and columns. Cancelation tests are most frequently used to detect USN and are more sensitive for this purpose 24,32,33. Line bisection tasks involve marking the midpoint of one or more horizontal lines and may involve lines of different lengths 29. Individuals with left neglect tend to make errors in the area to the right of true center. The average deviation from the center in the LB test in this study was 9.5 mm 24. Facchin et al. reported that the reliability of the LB test is medium to high and decreases slightly as the line length increases 28. These results are largely in agreement with other bisection tasks in healthy subjects. A greater deviation indicates USN, and we should use this measure as a complementary tool for diagnosing USN. During clinical practice, we should not apply only a single test to diagnose spatial neglect 33,34.

**Figure 1:**
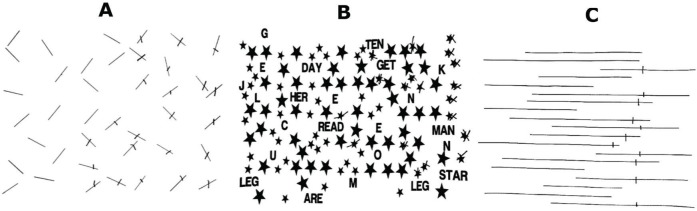
Clinical use of the three standardized neglect tests in a patient with USN (A) omission of the lines on the left side in LC; (C) omission of the smalls stars on the left side in SC; (C) right deviation of midline in LB.

Different USN tests can activate different cortical processes. Individuals who have problems on the LB task have more posterior lesions, such as lesions in occipitotemporal extrastriate areas 35. Verdon et al. found that lesions in the right inferior parietal lobule were more often associated with problems on the LB task, and lesions in the right dorsolateral prefrontal cortex were more often associated with problems on cancelation tasks 36. However, other authors have concluded that USN is usually associated with right parietal damage to the angular gyrus and can be tested for in clinical settings with both cancelation and LB tasks 24,. All three tests are useful for diagnosing USN in clinical practice. Figure 1 shows an example of the clinical use of three standardized tests on a patient who experienced a stroke in the right parietal lobe, with omissions in the left hemifield and midline deviation.

In this study, we observed that age is the main factor affecting performance in the LC and SC tests. The older the patient is, the worse his or her performance on the tests is likely to be. Several studies have reported factors that may affect USN test performance, and age is a well-discussed factor in the literature 24,40. A study conducted at Johns Hopkins Hospital that aimed to correlate age with USN test performance in ischemic stroke patients found that USN occurs more frequently in elderly individuals regardless of the size of injury or the severity of neurological symptoms 8. One of the proposed hypotheses is that older individuals have more serious attention deficits and decreased neural adaptivity following central nervous system injury. In addition, total brain volume tends to decrease with age, which may lead to cognitive impairment 41,42.

In our study, we observed that the number of omissions in the LC and SC tests showed no association with any demographic factors except for age, and the LB deviation was the smallest among individuals with the highest education levels. Azouvi et al. observed that in neurologically healthy individuals, older age and lower education level can lead to more errors in USN tests. Level of education affected the side on which omissions occurred 43. Specifically, individuals with higher education levels made more mistakes on the right, and individuals with less schooling made more mistakes on the left. It is possible that these differences are due to students being taught to write from left to right, making it more likely that individuals with high levels of education have reduced omissions on the left side 24,44,45.

The LB test is scored by measuring the deviation of the bisection from the true center of the line. Schenkenberg et al. defined a deviation of more than 6 mm from the midpoint as diagnostic of USN, which agrees with the data of this study 11. In the LB test, the main confounding factor related to deviation from center was education level. Azouvi et al. reported that factors such as education, age, and dominant hand should be considered in the diagnosis of USN 43. In a meta-analysis of the LB test, the authors concluded that young people make mistakes to the left, while older individuals tend to err to the right of center. There is inconsistency in the reports on the influence of sex on deviations from the midline. Different stages of the menstrual cycle have modulating effects on the location of the sagittal-median plane in women, but there are no significant reports on sex differences 24,. Chen et al. observed that women make leftward “where” spatial errors regardless of their age. These results point to sex-specific changes in the function of dorsal, cortical–cortical visuospatial networks in aged men compared to women 10.

The main limitations of the study relate to the testing of individuals within a single center. We also did not compare our results to those in the literature obtained using other existing assays, such as the Behavior Inattention Test, which uses 6 tests and is the gold standard for the detection of USN 24,27. Our aim was to establish norms and study practical and rapidly implementable tests that could be useful in clinical practice to facilitate the timely diagnosis of USN in acute neurological conditions arising from stroke, tumors, or trauma 24. A strength of this study is that it aims to motivate health professionals to consider USN in clinical evaluation, which could guide clinical approaches.

Based on our results, we can conclude that there are different cutoff points for USN diagnosis based on age: LC test >0 omissions regardless of age; SC test >0 omissions in individuals aged 18 to 35 years old, >2 omissions in individuals aged 36 to 60 years old, >3 omissions in individuals above 60 years old, and >1 omission in general; LB test >8 mm in individuals aged 18 to 60 years old, >13 mm in individuals aged 61 to 65 years old, >17 mm in individuals above 65 years old, and >9.5 mm in general. The cutoff points presented in this study may be indicative of USN, but due to performance differences based on age, we suggest using different norm scores for different age groups. These norm scores can be used in the clinic immediately for USN diagnosis.

## AUTHOR CONTRIBUTIONS

Luvizutto GJ was responsible for the data collection, processing, writing, literature search and critical review. Fogaroli MO, Theotonio RM, Neto EM were responsible for the data collection, processing and writing. Nunes HRC was responsible for the concept development, supervision and analysis. Bazan R was responsible for the concept development, supervision and critical review.

## Figures and Tables

**Table 1:** Demographic characteristics and performance of subjects in USN testing (n=150).

Variable	Summary	IQR
**Demographic variables**		
Sex		
Male : Female	76 (51%) : 74 (49%)	
Age (years)^(1)^	31.5 (18-87)	43.0
Caucasian : Non-Caucasian	112 (75%) : 38 (25%)	
Years of Education^(1)^	11 (0-16)	12
**USN performance**		
Line Cancelation Test		
Total number of omissions		
0	143 (95.3%)	
1	6 (4.0%)	
4	1 (0.7%)	
Omission (Total of lines not canceled >0)	7 (4.7%)	
**Star Cancelation Test**		
Total number of omissions		
0	123 (82.0%)	
1	16 (10.6%)	
2	6 (4.0%)	
>2	5 (3.4%)	
Omission (Total of stars not canceled >0)	27 (18.0%)	
Line Bisection Test		
Center deviation (MVD in mm)^(1)^	6.2 (2.1-6.6)	5.5

Summary in median (min-max); USN: unilateral spatial neglect; MVD: mean value of deviation; IQR: interquartile range.(1) Data expressed as median (minimum - maximum).

**Table 2:** Estimated odds ratios for the numbers of omissions in the LC and SC based on demographic variables.

	LC		SC	
Variable	OR (95% CI)	*p*	OR (95% CI)	*p*
Sex (male)	7.9 (0.6-99.5)	0.109	1.01 (0.34-2.00)	0.983
Age (years)	1.1 (1.02-1.2)	0.012	1.07 (1.03-1.11)	0.000
Years of education	1.0 (0.7-1.4)	0.889	0.88 (0.75-1.02)	0.094

LC=line cancelation; SC=star cancelation; OR=odds ratio; CI=confidence interval.

**Table 3:** Association between deviations from the center obtained in the line bisection and demographic variables.

Variable	Summary	*p*
Age (years)	r=0.16	0.052*
Years of Education	r=-0.20	0.015*
Sex^(1)^		
Female (n=76)	6.3 (3.0-38.3)	0.955**
Male (n=74)	6.1 (2.1-23.8)	

(1) Median (min-max);

* Spearman’s correlation.

** Mann-Whitney test.

**Table 4:** Descriptive table with the results of each USN test expressed in terms of mean, standard deviation and percentiles and stratified by age.

	LC			SC			LB
Age (years)	Average	SD	%	Average	SD	%	MVD
18-24 (n=20)	40.0	0	100	56.0	0	100	7.7
25-30 (n=21)	40.0		100	56.0	0	100	7.7
31-35 (n=14)	40.0	0	100	56.0	0	100	7.6
36-40 (n=16)	39.93	0.37	99.8	53.63	1.17	99.31	6.4
41-45 (n=18)	39.93	0.19	99.9	53.63	1.20	99.29	8.5
46-50 (n=16)	39.93	0.19	99.9	53.63	1.20	99.29	8.5
51-60 (n=14)	39.93	0.38	99.8	53.63	1.19	99.28	8.3
60-65 (n=15)	39.93	0.38	99.8	53.1	1.44	98.33	13.8
>65 (n=16)	39.91	0.42	99.6	53.0	1.53	98.21	17.6

Legends: LC=line cancelation test; SC=star cancelation test; LB=line bisection test; average of cancelations; SD=standard deviation; %=percentage of correct targets; MVD=mean value of deviation.

**Table 5:** Norm scores for line cancelation, star cancelation and line bisection tests in a Brazilian population, stratified by age.

			USN Tests							
			LC		SC		LB	LC	SC	LB
Age (years)	n	RW	Correct targets	Omissions	Correct targets	Omissions	MVD	RW*Omissions	RW*Omissions	RW*MVD
18-24	20	0.13	40	0	56	0	7.7	0	0	1.0
25-30	21	0.14	40	0	56	0	7.7	0	0	1.1
31-35	14	0.09	40	0	56	0	7.6	0	0	0.7
36-40	16	0.11	40	0	54	2	6.4	0	0	0.7
41-45	18	0.12	40	0	54	2	8.5	0	0	1.0
46-50	16	0.11	40	0	54	2	8.5	0	0	0.9
51-60	14	0.09	40	0	54	2	8.3	0	0	0.8
61-65	15	0.10	40	0	53	3	13.8	0	0	1.4
>65	16	0.11	40	0	53	3	17.6	0	0	1.9
Total	150	1.00					**Overall**	**0**	**1**	**9.5**

Legends: RW=relative weight (obtained by dividing the sample size of a specific range by the total sample size); LC=line cancelation test; SC=star cancelation test; LB=line bisection test; average of cancelations; MVD=mean value of deviation.

• For USN diagnosis, we recommended the following: ° LC test >0 omissions regardless of age. ° SC test >0 omissions (18 to 35 years old), >2 omissions (36 to 60 years old), >3 omissions (>60 years old), and >1 omission in general. ° LB test >8 mm (18 to 60 years old), >13 mm (61 to 65 years old), >17 mm (>65 years old), and >9.5 mm in general.
